# A Web-Based Data-Querying Tool Based on Ontology-Driven Methodology and Flowchart-Based Model

**DOI:** 10.2196/medinform.2519

**Published:** 2013-10-08

**Authors:** Xiao-Ou Ping, Yufang Chung, Yi-Ju Tseng, Ja-Der Liang, Pei-Ming Yang, Guan-Tarn Huang, Feipei Lai

**Affiliations:** ^1^Department of Computer Science and Information EngineeringNational Taiwan UniversityTaipeiTaiwan; ^2^Department of Electrical EngineeringTunghai UniversityTaichungTaiwan; ^3^Graduate Institute of Biomedical Electronics and BioinformaticsNational Taiwan UniversityTaipeiTaiwan; ^4^Department of Internal MedicineNational Taiwan University Hospital and National Taiwan University College of MedicineTaipeiTaiwan; ^5^Department of Electrical EngineeringNational Taiwan UniversityTaipeiTaiwan

**Keywords:** electronic medical records, query languages, information retrieval query processing, ontology engineering, clinical practice guideline

## Abstract

**Background:**

Because of the increased adoption rate of electronic medical record (EMR) systems, more health care records have been increasingly accumulating in clinical data repositories. Therefore, querying the data stored in these repositories is crucial for retrieving the knowledge from such large volumes of clinical data.

**Objective:**

The aim of this study is to develop a Web-based approach for enriching the capabilities of the data-querying system along the three following considerations: (1) the interface design used for query formulation, (2) the representation of query results, and (3) the models used for formulating query criteria.

**Methods:**

The Guideline Interchange Format version 3.5 (GLIF3.5), an ontology-driven clinical guideline representation language, was used for formulating the query tasks based on the GLIF3.5 flowchart in the Protégé environment. The flowchart-based data-querying model (FBDQM) query execution engine was developed and implemented for executing queries and presenting the results through a visual and graphical interface. To examine a broad variety of patient data, the clinical data generator was implemented to automatically generate the clinical data in the repository, and the generated data, thereby, were employed to evaluate the system. The accuracy and time performance of the system for three medical query tasks relevant to liver cancer were evaluated based on the clinical data generator in the experiments with varying numbers of patients.

**Results:**

In this study, a prototype system was developed to test the feasibility of applying a methodology for building a query execution engine using FBDQMs by formulating query tasks using the existing GLIF. The FBDQM-based query execution engine was used to successfully retrieve the clinical data based on the query tasks formatted using the GLIF3.5 in the experiments with varying numbers of patients. The accuracy of the three queries (ie, “degree of liver damage,” “degree of liver damage when applying a mutually exclusive setting,” and “treatments for liver cancer”) was 100% for all four experiments (10 patients, 100 patients, 1000 patients, and 10,000 patients). Among the three measured query phases, (1) structured query language operations, (2) criteria verification, and (3) other, the first two had the longest execution time.

**Conclusions:**

The ontology-driven FBDQM-based approach enriched the capabilities of the data-querying system. The adoption of the GLIF3.5 increased the potential for interoperability, shareability, and reusability of the query tasks.

## Introduction

A substantial number of health care records are routinely accumulated in clinical data repositories because of the increasing use of electronic medical record (EMR) systems. Previous studies have shown that these large volumes of clinical data offer great potential for discovering new knowledge and improving the quality of health care [1,2].

Experts from various domains can apply a query approach to elucidate the distributions of complex data by formulating and executing queries used for identifying the desired data that are stored in large clinical data repositories. Previous studies have proposed several query approaches based on specific query tasks that assist domain experts in retrieving clinical data for further analyses [3-5].

Extant literature on data querying from clinical data repositories are based on the following three considerations: (1) the interface design used for query formulation, (2) the representation of query results, and (3) the models used for formulating query criteria.

Regarding the design of a user interface for formulating queries, users can formulate queries by employing low-level query languages, such as Structured Query Language (SQL), or by using query-building tools that allow them to create query tasks easily using the features available with these tools.

The application of low-level query languages, such as SQL, presents several potential difficulties. First, experience in database querying is required. The users who employ SQL commands to query a database directly must possess a detailed understanding of the information in that database, including the table definitions, the table column types, and the relationships among the tables. Second, the SQL command syntax for complex queries could be difficult to write. When data querying involves a query algorithm, the SQL command syntax is complex, which can make it difficult to analyze the query results of these intermediate processes.

To minimize the complexity of formulating queries by using SQL commands, researchers have proposed and developed specific query-building tools to assist users in building and executing database queries. For example, RetroGuide was proposed to assist users who had limited database experience in formulating database queries [5]. The query task is formulated based on a flowchart by using the workflow editor, and the query criteria are specified in the nodes of the flowchart.

Regarding the representation of query results, various formats can be employed to present the queried data, including free text, structured tables, and visual charts. Rich data representation methodologies can assist in improving the users’ understanding of the database query results.

RetroGuide provides a table-based three-level hierarchical report of query results, including a summary report, detailed report, and information view of each patient [6]. Mabotuwana and Warren introduced a prescription timeline visualization function that allows clinicians to monitor the prescription situations of their patients by using graphical timeline charts [3].

Regarding the models used for formulating the database query criteria, the adoption of interoperable formulation information models has improved the opportunity for users to consistently query various clinical data repositories. Moreover, information models that allow powerful expression for query criteria formulation can enhance the capabilities of database queries.

Austin et al developed an information model for designing generic interfaces for EMR systems [4]. They collected a diverse set of examples of clinical questions that could be applied to represent database queries. Based on the queries, they identified several general patterns and designed an information model that represented clinical research queries.

Ontology-based approaches have been widely employed in various medical domains [7-10]. Mabotuwana and Warren proposed an ontology-driven [11] approach to formulate specific query criteria for enhancing the query capabilities of general practice medicine to improve the management of patients with hypertension.

In this study, we developed a Web-based approach for enriching the data query based on the three considerations discussed above. A prototype system was developed to test feasibility of applying a methodology for building a query execution engine using flowchart-based data-querying models (FBDQMs) by formulating query tasks using the existing Guideline Interchange Format (GLIF). The FBDQM was introduced, developed, and implemented in formulating query tasks by employing the flowchart and objects defined in the GLIF3.5 [12]. A graphical user interface that allows users to select entire or partial query criteria from a predefined query task, execute the formulated query, and present the query results was developed. The method proposed in this study could assist users with limited database experience in querying medical data. In addition to assisting users in formulating query tasks by using the flowchart-based models, the proposed approach involves employing a visual graphical interface for presenting the query results. The GLIF3.5 enhances the capability of queries, thereby increasing the potential for interoperability through the relevant standardized medical schemes involved in the GLIF3.5 (see Multimedia Appendices 1 and 2).

## Methods

### Overview of Methods

**Figure 1:**
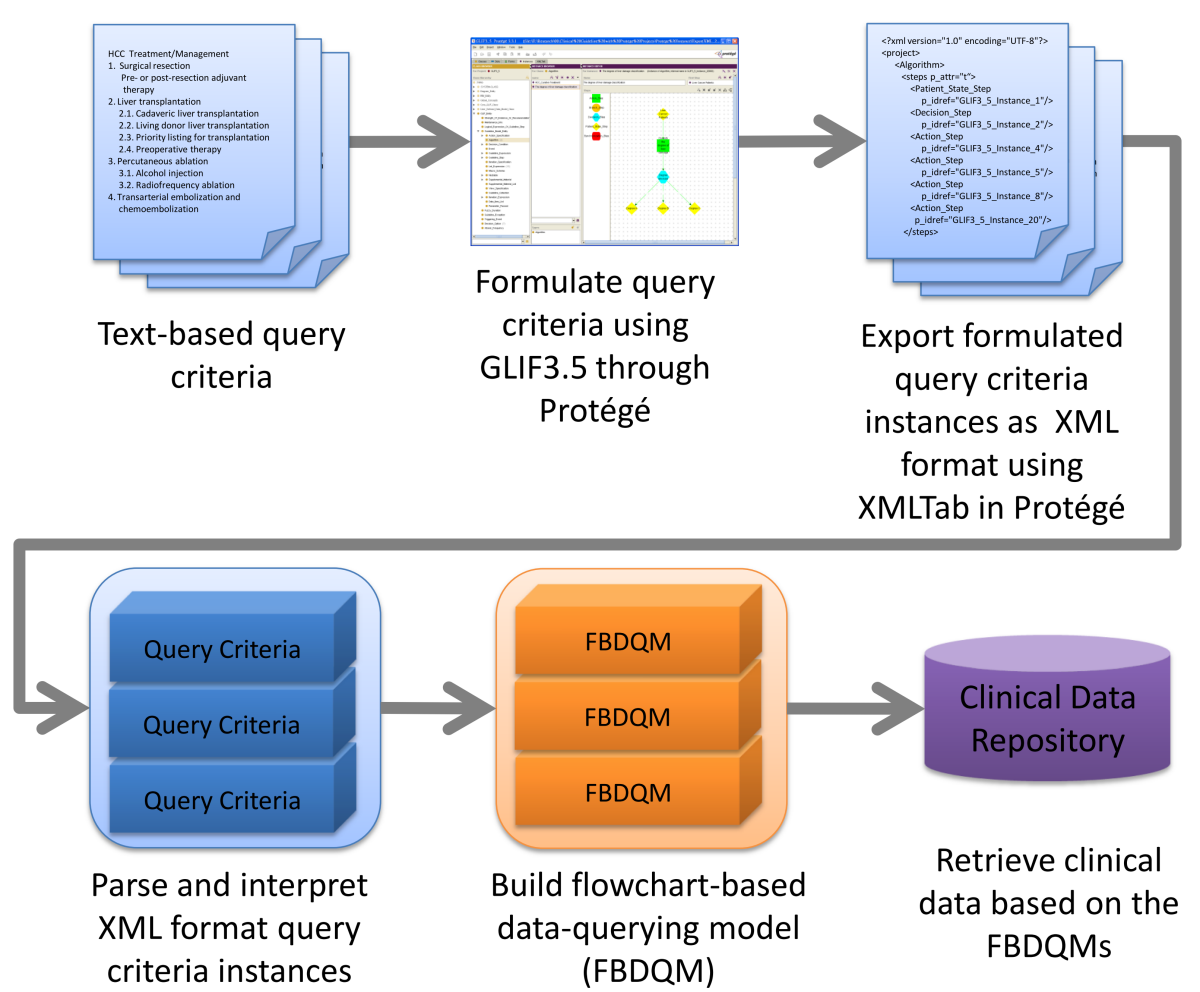
The overview of the methodology used in the data-querying tool based on ontology-driven methodology and flowchart-based model.

Figure 1 presents an overview of the methods proposed in this study. The text-based query tasks were formulated using the GLIF3.5 through the Protégé editing environment [13-15]. A native Protégé plug-in tool was used to export the formulated query tasks as Extensible Markup Language (XML) files. Subsequently, the XML files were imported into the proposed FBDQM-based query execution engine. The proposed engine interpreted the XML-formatted query tasks, executed the query operations, retrieved the clinical data, and displayed a representation of the query results.

### Clinical Practice Guideline Representation Languages

Clinical guideline representation languages were developed for formulating paper-based clinical guidelines in a computer-interpretable format. Currently, several medical-related institutions are developing numerous clinical practice guideline representation languages and models, including Asbru, EON, GLIF, GUIDE, PRODIGY, and PROforma [16,17]. The clinical guideline representation languages could be suitable for representing medical information in various computer-interpretable formats, including logic, criteria, and data items. Therefore, an existing clinical guideline representation language was employed to formulate the query criteria and query task items. The GLIF3.5 was selected to formulate the query tasks because a GLIF framework includes a set of steps that link together to form a flowchart [18]. The format of a flowchart can be employed to formulate the workflow of the query tasks, and complex query task workflows can be divided into several multistep subquery tasks. The GLIF model is object oriented, and XML-based syntax is used to present the class and instances of the class [18]. After formulating a GLIF-based query task, the query task can be translated into XML format for further interpretation. GLIF provides various abstraction levels. At the conceptual level, the rules and logic are represented as a flowchart, which allows users to formulate an overview of a query task before specifying all necessary detailed information. The rules and logic can be further specified at the computable level.

### Query Task Formulation

To formulate the query tasks using GLIF3.5, the concepts and criteria of the text-based query tasks should be categorized as the corresponding classes of the GLIF3.5 ontology. The *algorithm* class in GLIF3.5 is a flowchart that is used for describing the clinical guideline workflow. In this study, the flowchart was used for presenting the query task workflow.

To formulate the query tasks, users must employ Protégé, a knowledge-based editing software that provides a graphical user interface for the formulation of query tasks based on the GLIF3.5 flowchart. The query tasks are formulated by building the flowchart and specifying the criteria and data items in each node of the flowchart through the Protégé environment (see Multimedia Appendix 3).

The entire query task can be separated into numerous subtasks, and each subtask can be represented using a node in the flowchart. The following five predefined GLIF3.5 classes were used: (1) action, (2) decision, (3) branch, (4) synchronization, and (5) patient state. The detailed components of each node were further specified through Protégé based on the predefined GLIF3.5 ontology.

After using the GLIF3.5 ontology to formulate the query tasks, the nodes were exported from Protégé in the XML format and subsequently imported into the query execution engine for data query and retrieval of the clinical data repository (see Multimedia Appendix 4).

### System Architecture

**Figure 2:**
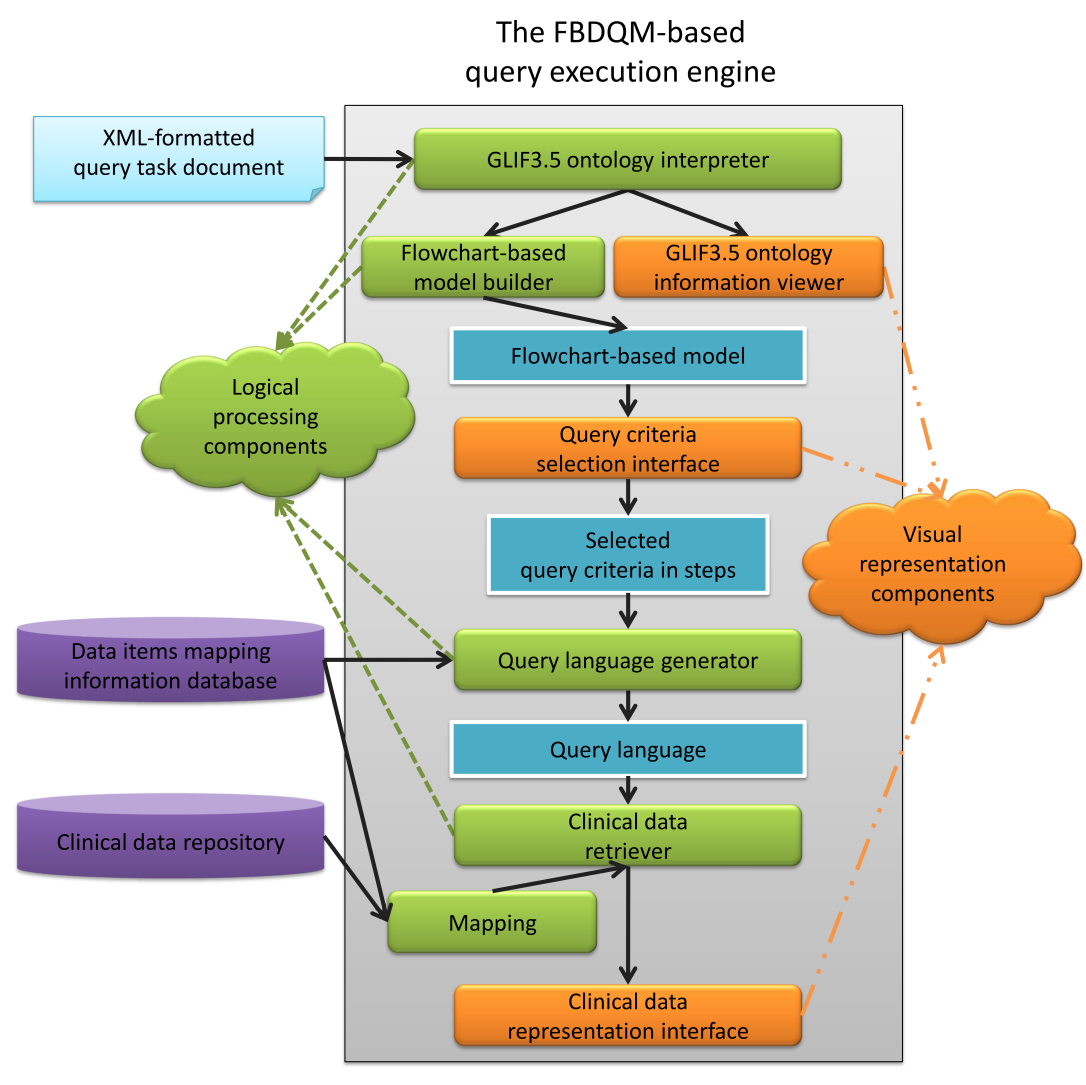
The architecture of the FBDQM (flowchart-based data-querying model)-based query execution engine.

A Web-based data-querying tool was implemented and the clinical data were queried using the FBDQM-based query execution engine. The architecture of the query execution engine comprises eight major components (Figure 2). These eight major components can be further separated into the following two sets: (1) logical processing components and (2) visual representation components.

The set of logical processing components includes the GLIF3.5 ontology interpreter, flowchart-based model builder, query language generator, clinical data retriever, and mapping component. The set of visual representation components contains the GLIF3.5 ontology information viewer, query criteria selection interface, and clinical data representation interface.

### GLIF3.5 Ontology Interpreter

A query task is formulated using the predefined GLIF classes. A GLIF3.5 ontology interpreter is necessary for parsing the GLIF-based query tasks and translating them into data query components for data retrieval. For example, following the interpretation process, a flowchart-based model builder was employed to create a flowchart-based model. A flowchart was displayed in the query criteria selection interface to provide an overview of the query task, and additional relevant information (eg, the criteria and data items in each node of the flowchart) could be viewed in the GLIF3.5 ontology information viewer.

The formulated query tasks were exported as XML-formatted documents and subsequently imported into the GLIF3.5 ontology interpreter in the query execution engine. The GLIF3.5 ontology interpreter was employed to interpret the query criteria and data items in the following five classes: (1) action, (2) decision, (3) branch, (4) synchronization, and (5) patient state. The original meanings of these classes in GLIF3.5 and their usages in this study are detailed as follows [12]. An action class is used for indicating an action to be performed. For example, this class was employed to detail medically oriented actions, such as medical treatment strategies. When the concepts in the query operation are relevant to medically oriented actions, these concepts are detailed based on the attributes of the action class. A decision class is used for specifying the criteria of various choices in a decision point. The decision option has a condition value attribute used to describe the detailed criteria of an option. When a query task contains the decision point and requires various criteria to determine the corresponding query operations, the decision class is used. The branch and synchronization classes work together. These two classes are used to express multiple concurrent paths in a flowchart. The concurrent paths are separated from the branch class and combined in the synchronization class. These two classes are used for representing multiple concurrent paths in a query task. The patient state class comprises the two functions. It is used to detail the clinical state of a patient and as a flowchart entry point. When the concept and the rule included in the query operation are relevant to the patient’s status, the patient state class is used for detailing the status. This class can also be used for detailing the start status of the query task.

### Flowchart-Based Model Builder

**Figure 3:**
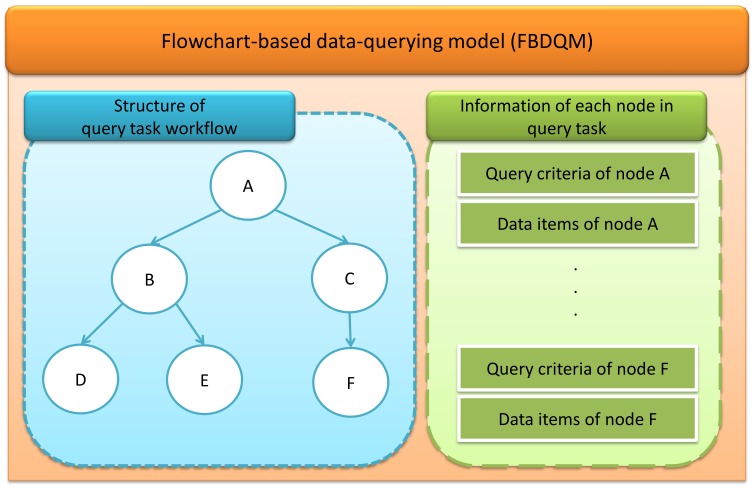
The flowchart-based data-querying model (FBDQM) containing the structure of query task workflow and the information of each node in the query task.

The flowchart-based model builder generates the flowchart-based model based on the interpreted results from the GLIF3.5 ontology interpreter. An interpreted query task is used to generate a corresponding FBDQM. The generated FBDQM contains the information relevant to the formulated query tasks, including the structure of the flowchart describing the overall query task and the detailed information of each query subtask, such as the query criteria and the related data items in each node (Figure 3). The FBDQM query tasks were derived primarily from instances of the algorithm class and objects related to the algorithm class.

A graphical flowchart of the FBDQM is used to present the workflow of a query task through the query criteria selection interface. Logical query criteria and relevant FBDQM data items are the inputs used by the query language generator to generate the corresponding query languages.

### Query Criteria Selection Interface

**Figure 4:**
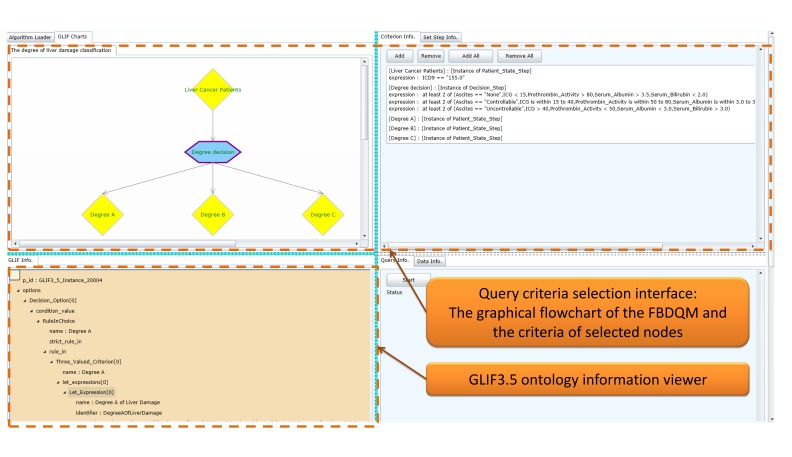
The query criteria selection interface and the GLIF3.5 ontology information viewer.

The query criteria selection interface in Figure 4 shows the graphical flowchart of the generated FBDQM. The query criteria selection interface provides flexibility in dynamically selecting all or certain flowchart nodes included in the execution of a query operation. All possible query elements were defined using GLIF through Protégé, and the user could subsequently select either specific or all of the criteria using the criteria selection interface. The query execution engine executes as many queries as the selected nodes in the flowchart that each node is essentially a separate query. For example, when a user queries all male patients with osteoporosis and a hip fracture [19], the criteria could be specified using a GLIF model containing various numbers of nodes (eg, patient state) to suit the needs of the user. A user could formulate a GLIF model with three nodes, where the first node contains a criterion for identifying gender, such as “gender=male,” the second node contains a criterion for identifying patients with osteoporosis, such as “ICD=733.00,” and the third node contains a criterion for identifying the patients with a hip fracture, such as “ICD=820.0.” A user could also formulate a GLIF model with only one node containing three criteria, such as “gender=male and ICD=733.00 and ICD=820.0.”

**Figure 5:**
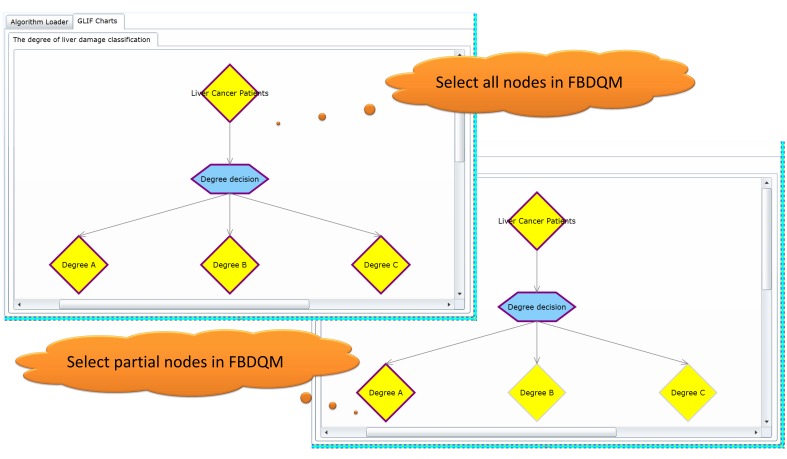
The flexibility in selecting all or partial nodes of the flowchart for participating in the execution of query operation.

**Figure 6:**
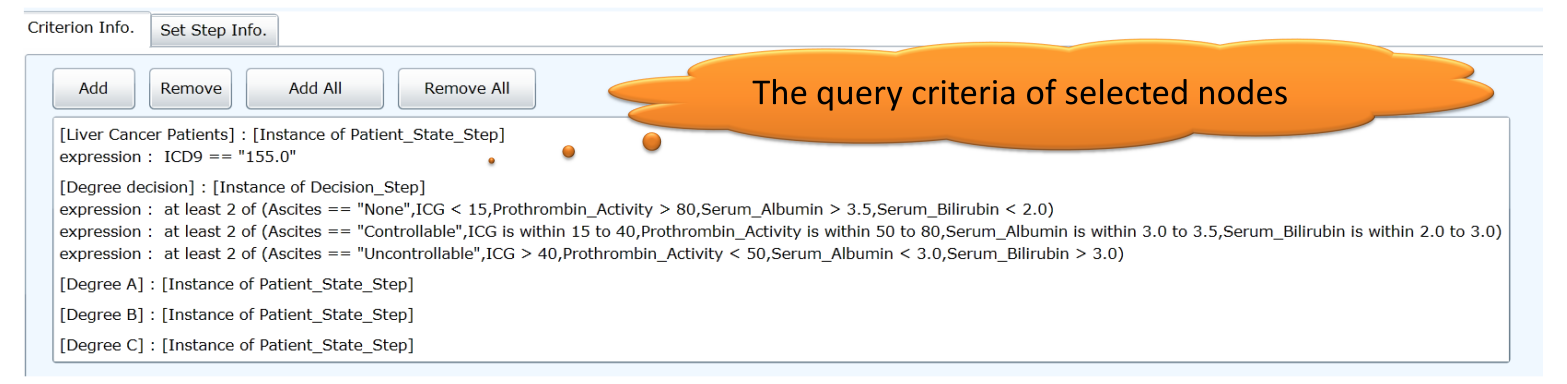
The query criteria of the selected nodes can be displayed in the query criteria selection interface.

The left side of Figure 5 shows that all nodes in the flowchart were selected for the query operation, and the right side of the figure shows that certain nodes in the flowchart were selected for the query operation. Figure 6 shows the query criteria of selected nodes that were displayed on the interface.

Furthermore, the query criteria selection interface provides the functionality of a mutually exclusive setting. For example, the degree decision node shown in Figure 5 comprises three child nodes (Degree A, Degree B, and Degree C). When a patient can only be classified into one of these three degrees, the degree decision node is set as a mutually exclusive node, and the priorities of its child nodes are set accordingly. Once the patient meets all the criteria of these three nodes, the patient is assigned to the node with the highest propriety.

### GLIF3.5 Ontology Information Viewer

Various interfaces were designed to provide multiple layers of views for the query tasks. An overview of the query tasks can be displayed in a flowchart in the query criteria selection interface. When a query task is highly complex, an overview of this query task can be viewed in a simplified flowchart. Each node of the flowchart contains a set of query criteria, and the detailed information (eg, the data items included in query criteria) can be viewed in the GLIF3.5 ontology information viewer by selecting the flowchart node.

**Figure 7:**
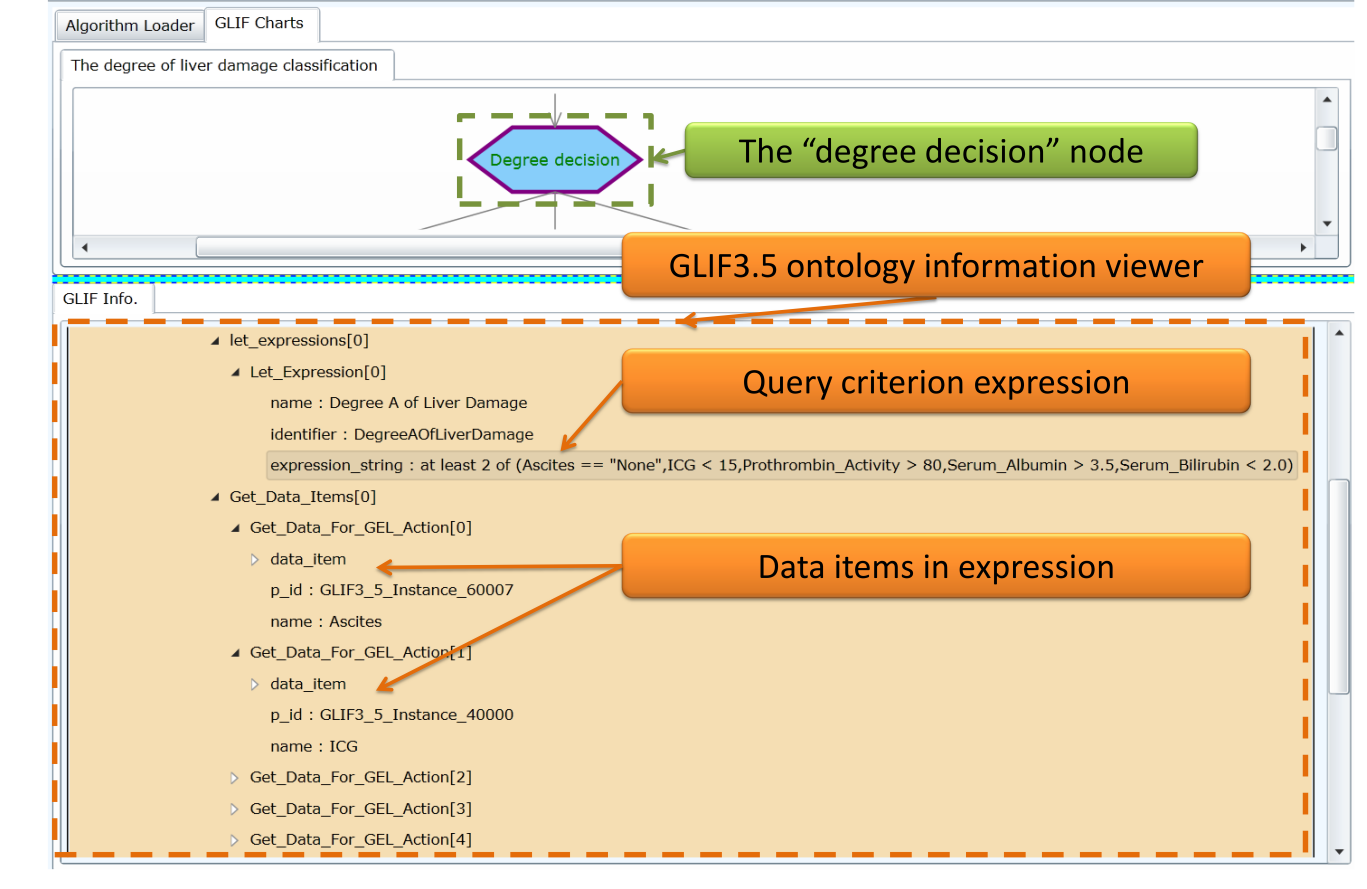
The GLIF3.5 ontology information viewer. The selected node, “degree decision,” and its corresponding information in GLIF3.5 format.

Figure 7 shows information relevant to the GLIF3.5 ontology in each node of the FBDQM presented in the GLIF3.5 ontology information viewer. When a specific node of the flowchart is selected, the detailed information of the GLIF3.5 ontology, including the name of the node, the query criterion expressions specified in the node, and the data items in the query criterion expressions, is displayed in the ontology information viewer.

### Mapping Component

The specific standards for medical terminologies and information models, such as vMR [20,21], are not assumed for using the GLIF to formulate query tasks. However, the GLIF provides the attributes for the encoders that specify further information such as the name, ID, and source of a concept (eg, name: cough, ID: C0010201, and source: UMLS). Therefore, to query the local clinical data repository, the mapping process is necessary for translating the concept specified in GLIF into the corresponding data in the local clinical data repository.

For example, as shown in Table 1, an ICD concept in the query task was mapped to the ICD9_Code from the diagnosis data table in the database. Laboratory items such as *Ascites* and *Prothrombin_Activity* were mapped to the corresponding data items with specific item names (ie, *Ascites* and *Prothrombin activity*) from the laboratory data table. Through the mapping process, a query language generator generates the corresponding SQL-based query languages to retrieve the corresponding data from the database.

To map the data items in a GLIF-formatted query task and those in a local clinical data repository, two mapping concepts proposed in the knowledge-data ontological mapper (KDOM) were employed [22]. The KDOM bridges the gap between the computer-interpretable guidelines encoded in the GLIF and the specific EMRs, and comprises the following four types of mapping between the guidelines and the medical records: (1) direct one-to-one field mapping, (2) temporal abstraction mapping, (3) classification hierarchy mapping, and (4) binary logical mapping. The concepts employed in this study were direct one-to-one field mapping and binary logical mapping. Direct one-to-one field mapping was implemented by predefining a mapping table, in which each record identified a single source data item encoded in the GLIF-formatted query tasks and a single destination data item in the local data repository. Binary logical mapping was implemented to manage complex query criteria, such as “at least condition.”

### Query Language Generator

The query criteria in the selected nodes from the FBDQM were transferred to the query language generator to generate the query language.

During the query language-generation process, the data items included in the query criteria of the selected nodes could be mapped onto the corresponding data items in the clinical data repository. In the query language generator, predefined mapping information is applied to map the data items. Mapping involves both the direct and indirect mapping. In direct mapping, the data items are mapped directly to the values of a specific column of a database table (eg, “select Personal_ID from Diagnosis where ICD9_Code=‘155.0’”; the data item was mapped directly to the value of the “ICD9_Code” column). In indirect mapping, the data items are mapped indirectly though multiple column values of a database table (eg, “select Personal_ID from Laboratory where Result_String=‘Controllable’ and Item_Name=‘Ascites’”; the data item was mapped indirectly though multiple column values, ie, the “Result_String” column and the “Item_Name” column).

After the data item mapping process is complete, the query language generator creates the corresponding SQL-based data query. The query language generator reads the query criterion in GLIF3.5 format and translates it into one or several simplified SQL criteria. Some examples of the query criteria in GLIF3.5 and the corresponding translated SQL queries are presented in Table 1. For example, the query criterion in GLIF3.5, “ICD9=155.0,” was translated into one SQL criterion, “select Personal_ID from Diagnosis where ICD9_Code= ‘155.0’.” The query criterion in GLIF3.5, “at least 2 of (Ascites==‘Controllable’, ICG is within 15 to 40, Prothrombin_Activity is within 50 to 80, Serum_Albumin is within 3.0 to 3.5, Serum_Bilirubin is within 2.0 to 3.0),” was translated and divided into five SQL criteria (Table 1).

To implement specific advanced queries to retrieve data from the repository, both SQL and high-level languages (ie, C#) were employed. For example, to implement the problem of “at least 2 of (Subcriterion 1, Subcriterion 2, Subcriterion 3, Subcriterion 4, and Subcriterion 5)” shown in Table 1, five subcriteria were implemented using SQL, and “at least 2” was further verified by implementing querying function in C#.

**Table 1:** The examples of the query criteria in GLIF3.5 and the corresponding translated SQL queries.

Query criteria format	Query criteria
GLIF3.5	1. ICD9=155.0
Translated SQL queries	1. select Personal_ID from Diagnosis where ICD9_Code=“155.0”
GLIF3.5	2. at least 2 of (Ascites==“Controllable,” ICG is within 15 to 40, Prothrombin_Activity is within 50 to 80, Serum_Albumin is within 3.0 to 3.5, Serum_Bilirubin is within 2.0 to 3.0)
Translated SQL queries	2.1. select Personal_ID from Laboratory where Result_String=“Controllable” and Item_Name=“Ascites” 2.2. select Personal_ID from Laboratory where Result_Nubmer between 15 and 40 and Item_Name=“ICG” 2.3. select Personal_ID from Laboratory where Result_Nubmer between 50 and 80 and Item_Name=“Prothrombin activity” 2.4. select Personal_ID from Laboratory where Result_Nubmer between 3.0 and 3.5 and Item_Name=“Serum albumin” 2.5. select Personal_ID from Laboratory where Result_Nubmer between 2.0 and 3.0 and Item_Name=“Serum bilirubin”

### Clinical Data Retriever

The clinical data retriever executes the query operation based on the query criteria in the selected nodes from the FBDQM. The query execution process commences from the nodes in the top layer of the flowchart and proceeds to those in the bottom layers. During query operation process in each node, the patients’ data are retrieved based on the translated SQL criteria, and the patients are reserved when they meet the query criteria specified in the node. The four types of notations that are used to represent the workflow and operations of the query execution in the FBDQM-based query execution engine are as follows: (1) QC(node), (2) PL(node), (3) PLS(node), and (4) PN(node).

QC(node) represents the query criteria included in the node. PL(node) constitutes the patient list, which contains the patients satisfying the query criteria included in the node. PLS(node) represents the size of the patient list, PL(node), indicating the number of patients who satisfy the query criteria included in the node. Finally, PN(node) is the list of the parent nodes of the node.

The left side of Figure 3 shows a flowchart comprising six nodes (nodes A, B, C, D, E, and F). Node A has two child nodes (nodes B and C), node B also has two child nodes (nodes D and E), and node C has one child node (node F). In the example, five nodes were selected for the query execution process (nodes A, B, C, D, and E). When the query operation was executed on one node, the patients in PL(PN(node)) were regarded as the query target set, and the query criteria contained in QC(node) were applied to PL(PN(node)). The query operation of the node was executed for patients who satisfied the query criteria of the parent nodes [ie, PL(PN(node))] with the query criteria of the node, QC(node). When a single node had no parent node, all patients in the clinical data repository were regarded as the query target set. For example, the first query operation, shown in Figure 3, was executed on node A, which had no parent node. The query execution of node A was based on QC(A), and QC(A) was applied to all patients in the repository. Following the first operation, the query result PL(A) was retrieved from the clinical data repository. The second query operation was executed using node B. The query execution on node B operated based on the patients in PL(A). The query criteria in QC(B) were applied to PL(A). For each patient in PL(A), when the patient satisfied all the query criteria in QC(B), that patient was retrieved and included in PL(B). Similarly, the query execution of node D was based on the patients in PL(B). The query criteria in QC(D) were applied to PL(B). Furthermore, the patient retrieved in the query operation of the lower layer node satisfied more query criteria (ie, that which satisfied the criteria in this layer node and its parent node) than the patient retrieved in the higher layer node (ie, that which satisfied the criteria in this higher layer node, but not the criteria in the lower layer node). Therefore, if the patient was included in PL(D), the patient must be included in both PL(B) and PL(A). PLS(D) was smaller than or equal to PLS(B), and PLS(B) was smaller than or equal to PLS(A). Similarly, PLS(C) was smaller than or equal to PLS(A). PLS(F) was zero because node F was not selected for the data query execution, and no patients were retrieved for this node.

### Clinical Data Representation Interface

**Figure 8:**
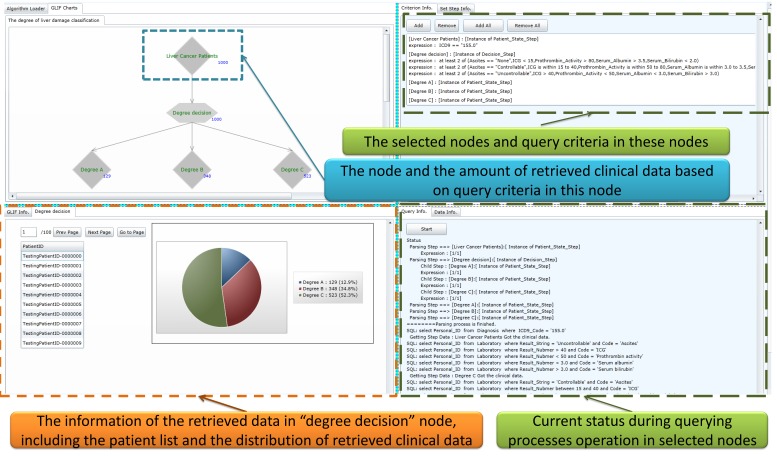
The clinical data representation interface.

The query results retrieved by using the FBDQM-based query execution engine in Figure 8 are represented using the following three formats: (1) the number of retrieved patients shown beside the nodes of the graphical flowchart, (2) the table-based patient list, and (3) the distribution information shown in the graphical pie chart.

**Figure 9:**
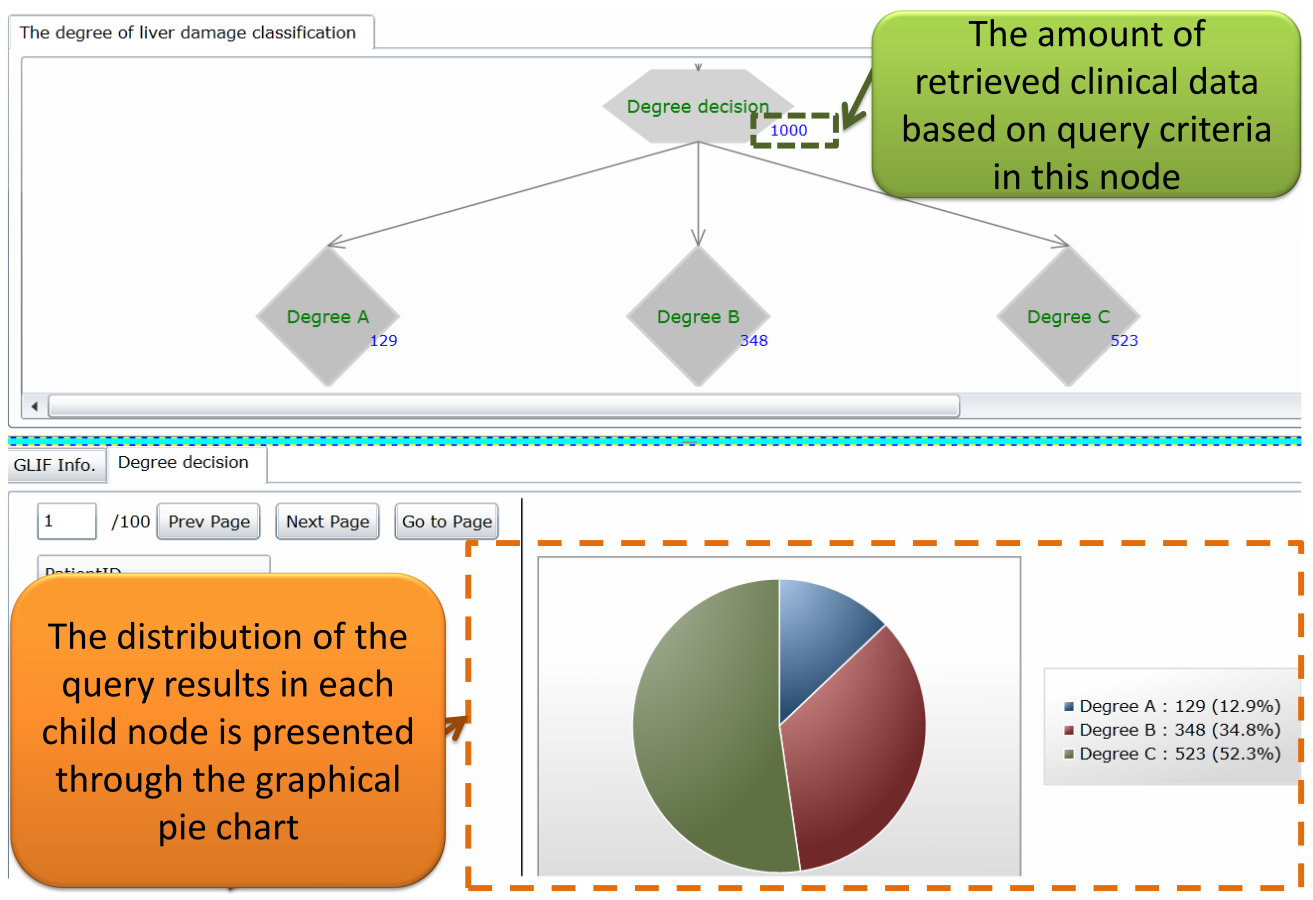
The clinical data representation interface showing the retrieved results of “degree of liver damage when applying a mutually exclusive setting” query task.

**Figure 10:**
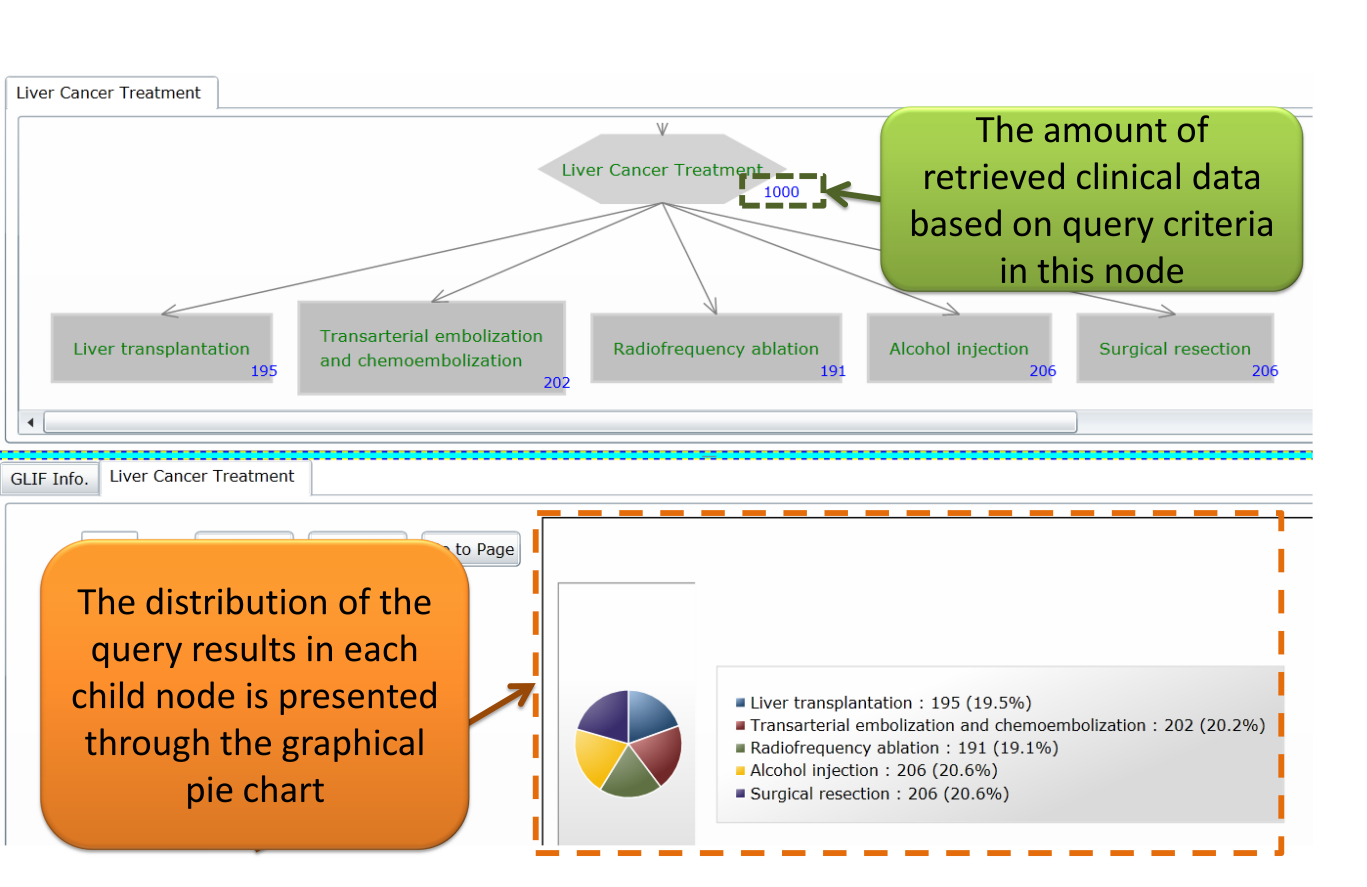
The clinical data representation interface showing the retrieved results of “treatments of liver cancer” query task.

Following the query execution of one node, the number of patients retrieved by the query operation is displayed dynamically beside the node in the flowchart. For example, in Figure 9, the number 1000 beside the degree decision node and the number 129 beside the Degree A node indicate the number of patients who satisfied the query criteria described in these nodes. The detailed query result of each node can be viewed upon the completion of the overall query executions of all nodes. The users can then select one node in the flowchart, and the detailed query result of that node, such as the table-based retrieved patient list of that node, is displayed. When a selected node has several branch child nodes, the distribution of the query results in each child node is presented using a graphical pie chart (Figures 9 and 10). For example, the distribution of the query results in the degree decision node is shown in the graphical pie chart in Figure 9 [ie, Degree A=129/1000 (12.90%), Degree B=348/1000 (34.80%), and Degree C=523/1000 (52.30%)].

### Evaluation of Functionality and Performance

The liver domain was selected because the clinicians who collaborated in this study are liver experts, and the related research topics are relevant to the treatment of liver cancer. The degree of liver damage is a critical factor in the selection of appropriate treatment strategies. Therefore, query tasks related to the treatment strategies of liver cancer and classifying the degree of liver damage were selected to evaluate the functionality and performance of the proposed system.

The accuracy and the time performance of the system were evaluated using three medical query tasks relevant to liver cancer based on the clinical data generator in the experiments with various numbers of patients (ie, 10 patients, 100 patients, 1000 patients, and 10,000 patients). Among the three query tasks, one was selected from the treatment strategy of liver cancer, and the remaining two were selected from the classification of the degree of liver damage [23-27].

To examine a broad variety of patient data, the clinical data generator is implemented to automatically generate the clinical data in the repository, and the generated data are employed to evaluate the system. The clinical data generator automatically creates the data items by randomly setting the values and subsequently storing data items, such as information regarding laboratory test results and treatment procedures, in the clinical repository. For example, the laboratory test results for Prothrombin_Activity were randomly selected from a predetermined range. The clinical data generator created the patients’ clinical data, including the diagnosis information, laboratory test results, and treatment procedures. Table 2 lists the distribution of the number of patients exhibiting various degrees of liver damage (ie, Degrees A, B, and C), both with and without applying the mutually exclusive setting. The distribution of the number of patients who received various treatments for liver cancer, including liver transplantation, transarterial embolization and chemoembolization, radiofrequency ablation, alcohol injection, and surgical resection, are also listed. The difference between the query tasks with and without applying the mutually exclusive setting is whether the system permits a patient to belong to more than one criterion among the set of criteria. When the mutually exclusive setting is applied in a query task for the degree of liver damage, a patient could only belong to one criterion among a set of criteria (ie, only Degree A, B, or C). When the mutually exclusive setting is applied, a summation of the number of patients belonging to three degrees is exactly equal to the total number of patients. For example, in a query task for the degree of liver damage in which the mutually exclusive setting was applied to a dataset of 100 patients, the total number of patients among the three degrees was exactly 100 (ie, the total number of patients in this dataset).

Three query tasks (degree of liver damage, degree of liver damage when applying a mutually exclusive setting, and treatments for liver cancer) were involved in the experiments. Both the degree of liver damage query task and the degree of liver damage when applying a mutually exclusive setting query task contained a total of 4 GLIF3.5-formatted query criteria, and a total of 16 translated SQL-formatted query criteria, whereas the treatments for liver cancer query task comprised a total of 6 GLIF3.5-formatted query criteria and 6 translated SQL-formatted query criteria.

**Table 2:** The distribution numbers of patients in four datasets that are randomly generated by the clinical data generator.

Dataset^a^	Degree of liver damage	Degree of liver damage when applying a mutually exclusive setting	Treatments for liver cancer
#1	Degree A: 1/10	Degree A: 4/10	LT^b^: 5/10
	Degree B: 3/10	Degree B: 7/10	TACE^c^: 2/10
	Degree C: 6/10	Degree C: 6/10	RFA^d^: 0/10
			AI^e^: 3/10
			SR^f^: 0/10
#2	Degree A: 11/100	Degree A: 52/100	LT^b^: 22/100
	Degree B: 36/100	Degree B: 60/100	TACE^c^: 23/100
	Degree C: 53/100	Degree C: 53/100	RFA^d^: 21/100
			AI^e^: 19/100
			SR^f^:15/100
#3	Degree A: 129/1000	Degree A: 555/1000	LT^b^: 195/1000
	Degree B: 348/1000	Degree B: 549/1000	TACE^c^: 202/1000
	Degree C: 523/1000	Degree C: 523/1000	RFA^d^: 191/1000
			AI^e^: 206/1000
			SR^f^: 206/1000
#4	Degree A: 1258/10,000	Degree A: 5298/10,000	LT^b^: 1984/10,000
	Degree B: 3409/10,000	Degree B: 5477/10,000	TACE^c^: 1970/10,000
	Degree C: 5333/10,000	Degree C: 5333/10,000	RFA^d^: 2079/10,000
			AI^e^: 1980/10,000
			SR^f^: 1987/10,000

^a^The datasets #1, #2, #3, and #4 are regarded as the datasets with different numbers of patients, including 10, 100, 1000, and 10,000 patients.

^b^LT: Liver transplantation.

^c^TACE: Transarterial embolization and chemoembolization.

^d^RFA: Radiofrequency ablation.

^e^AI: Alcohol injection.

^f^SR: Surgical resection.

## Results

### Experimental Results

In the experiments, the clinical data generator automatically generated various numbers of patients’ clinical data. Four datasets (ie, 10 patients, 100 patients, 1000 patients, and 10,000 patients) were generated randomly, and contained clinical data such as diagnosis data, laboratory test results, and treatment procedure data. The three query results of the three query tasks based on these four datasets were collected manually as the benchmark (ie, gold standard), against which the query results of the proposed system were compared to evaluate the accuracy of the proposed system. The accuracy of the three query tasks (ie, degree of liver damage, degree of liver damage when applying a mutually exclusive setting, and treatments for liver cancer) was 100% for all four experiments based on the four patient groups. This shows that the proposed system could perform all of the query operations accurately for the experiments.

Table 3 lists the time performance of the proposed system for the four experiments based on the three query tasks. The table shows the percentage of time taken to execute the entire query task. The total query execution time was divided into the following three phases: (1) “SQL operations”, (2) “criteria verification”, and (3) “other.” The SQL operations phase was the time taken to retrieve the data from the clinical data repository based on the translated SQL queries included in the entire query task. Criteria verification phase was the time taken to verify whether the retrieved patients’ data (which were retrieved using the translated SQL queries) met the query criteria defined in the nodes for the entire query task. For example, the five subcriteria of a query criterion, “at least 2 of (Subcriterion 1, Subcriterion 2, Subcriterion 3, Subcriterion 4, and Subcriterion 5)” shown in Table 1 were implemented using SQL, and “at least 2” was further verified by using the implemented querying function in C#. The “other” execution time was the time taken to parse the query criteria that were defined in the entire query task, translate the GLIF3.5 formatted query criteria into SQL queries, and set the query results in the representation interface. The total value was the total time taken to perform the entire query task. The total query operation time was divided into three phases (ie, SQL operations, criteria verification, and other) to evaluate the variance in time taken on these phases when the proposed system was applied to various datasets (ie, 10-10,000 patients). Table 3 shows the percentages for the distribution of the three phases and the execution time results (in seconds). Moreover, the figure shows the variances of time taken among the four experiments. For example, in the experiment in which the degree of liver damage of 10,000 patients was queried, 8.124 of the total execution time 36.666 seconds (22.16%) was spent executing SQL operations, 28.455/36.666 seconds (77.60%) was spent verifying the criteria, and 0.087/36.666 seconds (0.24%) was spent on other tasks.

**Figure 11:**
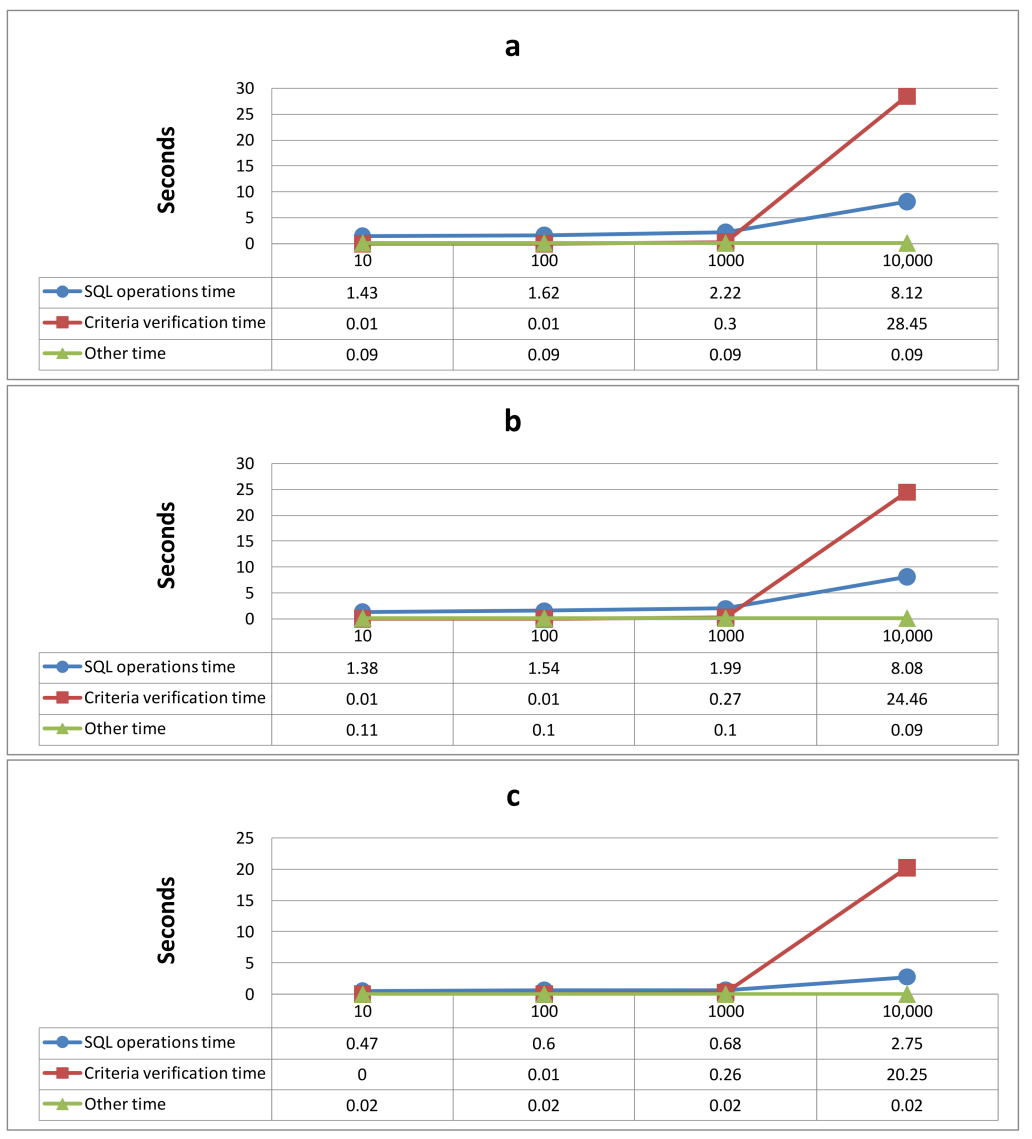
The performances of the system based on the three query tasks in four experiments with different number of patients, including a) degree of liver damage, b) degree of liver damage when applying a mutually exclusive setting, and c) treatments for liver cancer.

Figure 11 shows the performance of the proposed system based on the three query tasks in the four experiments involving various numbers of patients. The times taken for the SQL operations, criteria verification, and other tasks are represented by the three lines in Figure 11, and the execution time results (in seconds) are listed in Table 3.

### Software

The proposed system is an experimental version designed to test a novel methodology for building a query execution engine using FBDQMs by formulating query tasks using the existing GLIF. The proposed system was implemented based on Visual C# .NET, and Microsoft Silverlight technology was used to display the updated information dynamically during the data retrieval process. The system developed for this study is based on a Web-based architecture and is not provided as an open source. A new client-side user must install the Silverlight framework in the client-side computer to access the proposed system through a browser (eg, Internet Explorer or Google Chrome). For the server-side system, the database functions were provided by Microsoft SQL Server 2008. When the database was migrated (eg, from Microsoft SQL Server 2008 to Oracle), the programs relevant to data retrieval (eg, a program to retrieve data from a database based on specific SQL comments) must also be recoded.

**Table 3:** The performance in time of the system in experiment with three query tasks.

Item (patient number)	Degree of liver damage (seconds)	Degree of liver damage when applying a mutually exclusive setting (seconds)	Treatments for liver cancer (seconds)
SQL operations	93.82% (1.427)	92.60% (1.377)	95.76% (0.474)
Criteria verification	0.46% (0.007)	0.34% (0.005)	0.61% (0.003)
Other tasks	5.72% (0.087)	7.06% (0.105)	3.64% (0.018)
Total (10)	100% (1.521)	100% (1.487)	100% (0.495)
SQL operations	93.82% (1.623)	93.45% (1.542)	95.99% (0.598)
Criteria verification	0.75% (0.013)	0.55% (0.009)	0.96% (0.006)
Other tasks	5.43% (0.094)	6.00% (0.099)	3.05% (0.019)
Total (100)	100% (1.730)	100% (1.650)	100% (0.623)
SQL operations	85.13% (2.221)	84.22% (1.985)	70.90% (0.675)
Criteria verification	11.46% (0.299)	11.50% (0.271)	26.79% (0.255)
Other tasks	3.41% (0.089)	4.29% (0.101)	2.31% (0.022)
Total (1000)	100% (2.609)	100% (2.357)	100% (0.952)
SQL operations	22.16% (8.124)	24.75% (8.076)	11.95% (2.750)
Criteria verification	77.60%(28.455)	74.97% (24.461)	87.96%(20.248)
Other tasks	0.24% (0.087)	0.28% (0.092)	0.10% (0.022)
Total (10,000)	100% (36.666)	100% (32.630)	100% (23.020)

## Discussion

### Results of the Query Tasks

The results of the three query tasks show that when more query target patients are in the database, more total execution time is spent on the query operation. The phases that required the greatest length of execution time were the SQL operations and the criteria verification phases (Table 3).

The proposed system simplified complex GLIF3.5-formatted query criteria into one or more SQL-based units. The complicated query criteria was verified during the criteria verification process after the patient sets were retrieved based on the simplified SQL queries during the SQL query process. Therefore, the increase in the execution time for the criteria verification process was greater than that for the SQL query process when the total number of patients increased. Table 3 shows that more than 90% of the total execution time was spent performing the SQL query process in two experiments (ie, 10 patients and 100 patients). Compared with these two experiments, the percentage of the total execution time for the SQL query processes decreased, and the percentage of the execution time for the criteria verification process increased during the experiments involving 1000 patients and 10,000 patients. Figure 11 shows a greater increase in the time taken for the criteria verification process (the red lines with the square points in the three query tasks) than for the SQL query process (the blue lines with the circle points in the three query tasks) when the patient set is larger, especially in the experiment with 10,000 patients.

For the query tasks when applying the mutually exclusive setting, once the patients had been verified and had met the criteria for the one-child nodes, the patients did not require further verification for the other child nodes. In this situation, less time was required to perform the criteria verification process. Therefore, the query task with the mutually exclusive setting required less time than those without applying the mutually exclusive setting (Table 3).

### Advantages of the Approach

The approach proposed in this study provides several beneficial features. First, the adoption of GLIF3.5 increases the potential for interoperability and shareability of database queries. This study was inspired by the work of GLIF3.5 and employed GLIF3.5 classes such as “algorithm” to formulate the query tasks. Thus, the benefits provided by GLIF3.5 can be inherited. GLIF3.5 is a clinical guideline representation language that was originally developed for formulating and sharing computer-interpretable clinical practice guidelines. The concepts, patient data items, and query criteria in the query tasks can be represented using standard vocabularies, medical data models, and medical logical expression languages of criteria (eg, the Unified Medical Language System, UMLS; HL-7’s Reference Information Model version 1.0, RIM; and Guideline Expression Language, GEL [12,22,28]).

Second, GLIF3.5 contains flowchart-based models. The GLIF3.5 algorithm class is used to formulate the algorithm included in the clinical guidelines [12,22,28]. In the discussed RetroGuide, the flowchart-based query methodology is used to assist users with limited database experience in formulating the query tasks. In this study, the flowchart-based instances provided by GLIF3.5 were adopted to assist users in formulating the overall workflow of the query tasks. Each node in the flowchart was considered a subprocess of the overall query process. Third, this system provides a visual representation of the query results. The query results including the amount of patient data retrieved (shown beside the nodes of the graphical flowchart), the table-based patient list, and the distribution information (shown in the graphical pie chart) are presented on the visual graphical interface. Fourth, the query criteria selection interface provides the flexibility for users to select all or certain nodes in the flowchart to execute the query operation process. Finally, the formulated query tasks can be stored as a Protégé project file, thereby facilitating the reusability of the query tasks.

### Related Work

Austin et al in 2008 proposed a method for consistently querying one or more EMR systems based on many years of European research and standardization of the interoperable communication of EMRs [4]. Their work contributed to and highlighted the feasibility of standardizing query interfaces. However, this study did not focus on defining information models, but an existing model, GLIF, was employed for formulating the query tasks.

A year later, Mabotuwana and Warren proposed a tool for displaying the prescription information of patients by employing visual timeline graphical charts and applying an ontology-driven approach for formulating query criteria [3]. An ontology-driven approach was used for formulating the query criteria, and the query results were presented using graphical charts. However, unlike the visual timeline graphical charts of Mabotuwana and Warren, the query results in this study were not presented using temporal information. Their visualization of the timeline provided clear and rich information that was relevant to the prescription of a selected patient. In this study, the GLIF was applied to formulate the flowchart-based query tasks. A user can select the nodes in the flowchart for executing the query operation, and the flowchart showed the query results (eg, the number of patients).

In RetroGuide, a query task was formulated based on the flowchart using the workflow editor. Instead of employing medical-specific knowledge representation standards such as Asbru, EON, GLIF, and PROForma [29], the authors of RetroGuide employed a standard workflow definition language, XML Process Definition Language (XPDL), which was a cross-industry workflow technology, and they presented the possibility of applying various workflow engines or editors at different institutions. RetroGuide provided table-based three-level hierarchical reports of query results (ie, summary report, detailed report, and the information view of a patient). Previous research showed that numerous medical-specific knowledge representation standards have a considerable capability for modeling workflow and providing highly sophisticated medicine-specific modeling constructs [19].

Although the query language generator and the clinical data retriever employed in this study can retrieve the clinical data by interpreting GLIF-formatted query tasks using the GLIF3.5 ontology interpreter, the components in this study do not function as a regular guideline execution engine. The components developed in this study provide functions for querying the clinical data based on specific criteria, although they do not function as a guideline execution engine for updating the medical decisions determined by clinicians. There is a prior published report on GLIF execution engine called GLEE [30]. There are several differences between the components in this study (ie, in the FBDQM) and those in the GLEE. Primarily, the GLEE applies a specific guideline to a selected patient, presents optional steps for clinicians on the client side of the system, and waits for the clinicians to make their selections. Subsequently, the GLEE schedules the following steps and updates the relevant records. The primary purpose of the FBDQM is to query the patients’ data based on the user-specified query criteria.

Cohort identification is an essential process of clinical research. Previously, cohort identification approaches such as the informatics for integrating biology and the bedside (i2b2) hive [31] from the i2b2 group were proposed. The i2b2 hive is an open-source software platform that enables managing medical records and genomic data to facilitate research. The i2b2 hive comprises a set of modules that communicate based on Web services. The i2b2 hive is suitable for estimating cohort sizes and generating research cohorts through simple inclusion-exclusion criteria [32], and the query results can be represented using a timeline view. In our study, a flowchart-based method was proposed for formulating the workflow of query tasks. A complex query task was divided into several subquery tasks by applying various flowchart nodes, and the query results of these subquery tasks were shown in separate nodes. Therefore, a flowchart-based method provides a hierarchical view for displaying the query results through various hierarchical layers, implying that the patient data retrieved in the query operation of the lower layer node satisfied more query criteria (ie, satisfying the criteria in this layer node and its parent node) than the patient data retrieved in the higher layer node (ie, satisfying the criteria in the higher layer node, but not the criteria in the lower layer node). This flowchart-based method allowed the observation of variations among the query results in the different layers.

### Limitations

Although this system enriches the capability of data querying using the ontology-driven and FBDQM-based approaches, it does present several limitations. First, the query criteria in nodes cannot be directly modified or created using the criteria selection interface. The query criteria must be formulated in advance using the GLIF3.5 format in Protégé. Subsequently, these criteria are exported in the XML format and are managed by the proposed system. If the query criteria in the node require modification, or if new criteria must be added in the node, the criteria should be modified or created using the Protégé environment.

Second, to perform the data mapping, the data item mapping table should be predefined in the database. The query language generator used the predefined mapping information to map the data items. During the mapping process, the data items in the query criteria are mapped to the data items in the database. When the mapping information is not predefined in the database, the mapping process might be performed incorrectly.

Third, *one*-*item*-*to*-*one*-*item* mapping was supported in the study. In one-item-to-one-item mapping, one item within the query criteria is mapped to an item in the database. Other mapping types, such as *one*-*item*-*to*-*many*-*items* mapping and *many*-*items-to-one*-*item* mapping are not supported in this study. For example, in the data repository, the ICD9 codes could appear with various data sources in the database (eg, ICD9 codes for inpatients and ICD9 codes for outpatients). During the data-mapping process, the data-mapping table should only specify one source (ie, ICD9 codes for inpatients or ICD9 codes for outpatients) for mapping the ICD9 code values. The framework does support the idea of a valueset by indicating values using the “or” operator. For example, the framework does not support the ability to enumerate a list of ICD9 codes for a node such as “ICD=155.0 OR 155.2 OR 156.3,” although it supports the managing of a list of ICD9 codes specified as “ICD=155.0 OR ICD=155.2 OR ICD =156.3.”

Fourth, although the proposed approach could assist users with limited database experience in formulating the query tasks, they must understand how to employ the GLIF3.5 components to formulate the query tasks.

The fifth limitation is related to the problem of common representation for patient parameters (eg, diagnoses, procedures, demographic data, and laboratory results). In this study, the data formulated using the GLIF are not mapped to the common representations, such as vMR [20,21]. A common representation for patient parameters facilitates the interoperability of queries across various databases. In this study, a mapping process was included to map the query task data items to data items in a local database.

Sixth, GELLO is an object-oriented query and expression language [33]. In this study, GELLO was not involved in mapping query tasks and data items in the local data repository. Conversely, the SQL queries employed for the querying data processes in this study are generated directly according to the query criteria in the GLIF-formatted query tasks through the mapping and query language generator mechanism in the study.

Seventh, this study has a limited set of examples related to the liver disease domain, and it is not been tested in numerous domains. When a mapping table (ie, the source data item is mapped to a destination data item in the local database) for other disease domains is defined, the system could be capable of managing query tasks for other diseases.

The eighth limitation is that this query platform is focused only on the cohort estimation counts (ie, patient counts). The query platform can be used for collecting patient IDs (ie, cohort) but not for cohort data (eg, cancer cohort with data on tumor size, survival, and laboratory values) in the dataset results. For example, it can solve problems involving the number of patients in the database with liver tumors measuring 2 cm or smaller and a range of value of a specific laboratory, but not a dataset on all liver cancer patients with the data on tumor size and this specific laboratory result.

GLIF as format is not being actively improved; GLIF3.5 is the current version. Furthermore, because the interpreter developed in this study was based on the GLIF schema, the method proposed in this study is useful only for interpreting query tasks formulated using the GLIF. Query tasks based on the GLIF are created using an interface provided by Protégé. This system is a laboratory experiment for presenting the feasibility of the methodology presented in this study. Users of the proposed system are both creators and key collaborators.

### Future Work

This experiment was conducted to evaluate the feasibility of applying a methodology used for building a query execution engine by formulating query tasks using the existing GLIF. The framework can be used in clinical research when a researcher must identify patients based on specific criteria. The framework could be enhanced further by retrieving both cohort and patient data (eg, structured data and relevant clinical narrative reports). Furthermore, because the query tasks were formulated using clinical guideline representation language, the framework can also be used to verify the status of guideline compliance by querying the patient data using an encoded guideline.

### Conclusion

The FBDQM-based query execution engine comprises eight major components, including logical processing components and visual representation components. The proposed FBDQM-based query execution engine was implemented to interpret the XML-formatted query tasks that were formulated using GLIF3.5, execute the query operations, retrieve clinical data, and represent the query results. In the experiments involving different numbers of patients, the FBDQM-based query execution engine performed successfully in retrieving the clinical data based on the query tasks formatted using GLIF3.5.

The ontology-driven and FBDQM-based approach enriched the data query capabilities along the three major considerations: using the query interface for query task formulation, representing query results, and employing models to formulate query criteria. The potential for interoperability, shareability, and reusability of the query tasks was increased by adopting GLIF3.5.

## Multimedia Appendix 1

The video file for demonstrating the proposed approach.

## Multimedia Appendix 2

The link to YouTube video for the demonstration of the proposed study.

## Multimedia Appendix 3

More information related to the formulation of query tasks using GLIF3.5 through Protégé and the translation of query tasks into the XML format document.

## Multimedia Appendix 4

The XML file containing two examples of query tasks described in this paper. These query tasks are formulated using GLIF3.5 and translated into XML documents. These query tasks are only used for testing the performance of the proposed system and have not been validated medically by clinicians. They should only be seen as examples of query tasks encoded using GLIF3.5.

## Abbreviations

EMR: electronic medical record
FBDQM: flowchart-based data-querying model
GEL: Guideline Expression Language
GLEE: GLIF execution engine
GLIF3.5: Guideline Interchange Format version 3.5
KDOM: knowledge-data ontological mapper
RIM: Reference Information Model
SQL: Structured Query Language
UMLS: Unified Medical Language System
XML: Extensible Markup Language
XPDL: XML Process Definition Language
