# Supplementary material for: A Web-Based Data-Querying Tool Based on Ontology-Driven Methodology and Flowchart-Based Model
Source: JMIR Med Inform. 2013 Oct 8;1(1):e2. doi: 10.2196/medinform.2519 (PMC4288233; doi:10.2196/medinform.2519)

Please use the following YouTube link for viewing the video that is used for demonstrating the proposed approach in this study:
http://www.youtube.com/watch?v=lUvYbuIP7og
(720p HD may have better video quality)

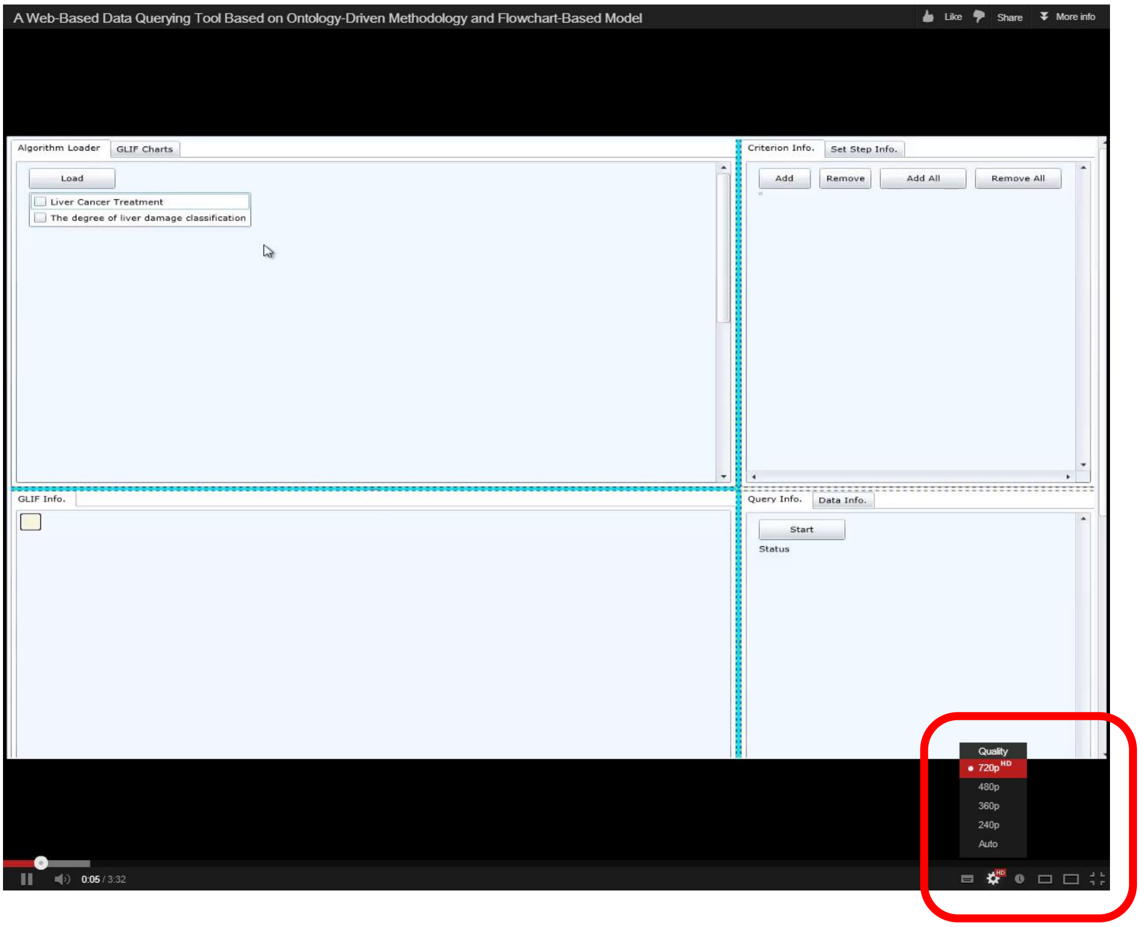

Supplement: Supplementary file 2 [file medinform_v1i1e2_app2.pdf]
