# Supplementary material for: A Web-Based Data-Querying Tool Based on Ontology-Driven Methodology and Flowchart-Based Model
Source: JMIR Med Inform. 2013 Oct 8;1(1):e2. doi: 10.2196/medinform.2519 (PMC4288233; doi:10.2196/medinform.2519)
Supplement: Supplementary file 3 [file medinform_v1i1e2_app3.pdf]

The formulation of query tasks using GLIF3.5 through Protégé and the translation of query tasks into the XML format document

To formulate the query tasks using GLIF3.5 [1], the concepts and criteria of the text-based query tasks should be categorized as the corresponding classes of the GLIF3.5 ontology. The algorithm class in GLIF3.5 is a flowchart used for describing the clinical guideline workflow. In this study, the flowchart was used for presenting the query task workflow.

The entire query task can be separated into numerous sub-tasks, and each sub-task can be represented using a node in the flowchart. The following five predefined GLIF3.5 *classes* were used: (a) *action*; (b) *decision*; (c) *branch*; (d) *synchronization*; and (e) *patient state*. The detailed components of each node were further specified through Protégé 3.3.1 based on the predefined GLIF3.5 ontology. After using the GLIF3.5 ontology to formulate the query tasks, they were exported from Protégé 3.3.1 in the XML format by using a native Protégé plug-in tool (*XML Tab*).

(Please note that if the following links do not work by directly clicking links in PDF, then please try the following methods: (a) copy and paste the full link to a web browser; (b) open this PDF using web browser by dragging this PDF into a web browser and then click the links in this PDF; or (c) use different version of PDF reader. Thanks)

GLIF ontology schema

GLIF3.5 ontology needs be prepared to formulate a query task using the classes in

GLIF3.5. A complete GLIF3.5 ontology schema can be obtained through the following link, GLIF3.5 ontology in Protege (The full link is http://mis.hevra.haifa.ac.il/~morpeleg/Intermed/guidelines/GLIF3_5.zip) (a file named "GLIF3_5.zip" can be downloaded). The link can be accessed through this web page, GLIF Ontology and Validation Tool (The full link is http://mis.hevra.haifa.ac.il/~morpeleg/Intermed/guidelines/Protege_Ontology.htm). This web page is under Professor Mor Peleg's website (The full link is http://mis.hevra.haifa.ac.il/~morpeleg/). "GLIF3_5.zip" provides full ontology schema of GLIF3.5 and can be viewed and used by opening the file named "GLIF3_5.pprj" (which is included in the "GLIF3_5.zip" file) through Protégé 3.3.1 (The full link is http://protege.cim3.net/download/old-releases/Protege%203.x/3.3.1/full/). Moreover, examples of GLIF3.4 also provided by this web page GLIF Ontology and Validation Tool. Please note the statements of **disclaimer** in this web page. (The full link is http://mis.hevra.haifa.ac.il/~morpeleg/Intermed/guidelines/Protege_Ontology.htm).

Brief descriptions of five major classes

GLIF3.5 has an abstract class *Guideline_Step* which includes five subclasses to formulate the algorithm of a clinical guideline (a query task in this study), including *action*, *decision*, *branch*, *synchronization*, and *patient state*. The classes are used for specifying the algorithms of the query criteria. The original meanings of these classes in GLIF3.5 and their usages in this study are detailed as follows [1]. An action class is used for indicating an action to be performed. For example, this class was employed to detail medically oriented actions, such as medical treatment strategies. When the concepts in the query operation are relevant to medically oriented actions, these concepts are detailed based on the attributes of the action class. A decision class is

used for specifying the criteria of various choices in a decision point. The decision option has a condition value attribute used to describe the detailed criteria of an option. When a query task contains the decision point and requires various criteria to determine the corresponding query operations, the decision class is used. The branch and synchronization classes work together. These two classes are used to express multiple concurrent paths in a flowchart. The concurrent paths are separated from the branch class and combined in the synchronization class. These two classes are used for representing multiple concurrent paths in a query task. The patient state class comprises the two functions. It is used to detail the clinical state of a patient and as a flowchart entry point. When the concept and rule included in the query operation is relevant to the patient's status, the class is used for detailing the status. The class can also be used for detailing the start status of the query task.

Specifications and further information related to GLIF

The specifications, tutorial slides, and related documents of GLIF can be obtained through Guideline Representation Page (The full link is http://mis.hevra.haifa.ac.il/~morpeleg/Intermed/GLIF.htm). These documents provide rich information related to the formulation of GLIF models. For example, GLIF 3.5 specification document (The full link is http://mis.hevra.haifa.ac.il/~morpeleg/Intermed/guidelines/GLIF_TECH_SPEC_May_4_2004.pdf) introduces methods for building a flowchart, definitions of five major classes of GLIF3.5 which are used to build a flowchart, and other related classes such as the specific classes used to formulate query criterion expressions (e.g., *criterion* class). The slides of GLIF tutorial (The full link is http://mis.hevra.haifa.ac.il/~morpeleg/Intermed/guidelines/GLIF_TECH_SPEC_May

_4_2004.pdf) can also be obtained through Guideline Representation Page (The full link is http://mis.hevra.haifa.ac.il/~morpeleg/Intermed/GLIF.htm).

Translation of query tasks into the XML format document

After using the GLIF3.5 ontology to formulate the query tasks, they were exported from Protégé 3.3.1 in the XML format by using a native Protégé plug-in tool (*XML Tab*). The information of XML tab can be obtained through a web page related to Protégé (The full link is http://protegewiki.stanford.edu/wiki/XML_Tab). The query task is included in the root element named *Project* in the XML document and the further content is included in the sub-elements of *Project* such as *Algorithm* and *Medically_Oriented_Action_Specification*. Moreover, an instance of GLIF model may be used by other instances, and reference numbers such as *p_id="GLIF3_5_Instance_ a serial number"* and *p_idref=" GLIF3_5_Instance_ a serial number "* are given for identifying a specific instance.

Reference

1. Boxwala AA, Peleg M, Tu S, Ogunyemi O, Zeng QT, Wang D, Patel VL, Greenes RA, Shortliffe EH. GLIF3: a representation format for sharable computer-interpretable clinical practice guidelines. J Biomed Inform 2004 Jun;37(3):147-61. PMID:15196480.
